# Longitudinal eosinophil‐derived neurotoxin measurements and asthma development in preschool wheezers

**DOI:** 10.1111/cea.14210

**Published:** 2022-08-13

**Authors:** Sandip Chakraborty, Katarina Stenberg Hammar, Anastasia E. Filiou, Idun Holmdahl, Angela Hoyer, Helena Ekoff, Anders Sjölander, Niclas Rydell, Gunilla Hedlin, Jon R. Konradsen, Cilla Söderhäll

**Affiliations:** ^1^ Department of Women's and Children's Health Karolinska Institutet Stockholm Sweden; ^2^ Astrid Lindgren's Children's Hospital Karolinska University Hospital Stockholm Sweden; ^3^ Thermo Fisher Scientific Uppsala Sweden

**Key messages**

- Increased EDN levels from age four were associated with asthma development in preschool wheezers.
- EDN is more closely associated with asthma development than blood eosinophils in preschool wheezers.
- At age seven, preschool wheezers with asthma had higher EDN levels than those without.

To the Editor,

Asthma is the most common chronic disease during childhood, often preceded by wheezing episodes during preschool age. Increased risk of wheeze has been associated with lower respiratory tract infections, day care attendance, postnatal smoke exposure, atopy and male gender. Recurrent wheeze in preschool children is a major clinical problem that requires high healthcare and economic resources. While most preschool wheezers outgrow their symptoms, more than one‐third have developed asthma by the age of six, and more than half by the age of 17.^1^, ^2^ Eosinophils are involved in the innate and adaptive immune systems and play an essential role in asthma and allergic diseases. Eosinophil‐derived neurotoxin (EDN), one of the major proteins in eosinophilic granules, has antiviral and antibacterial activity, and its release is triggered by pro‐inflammatory mediators.^3^, ^4^ Available information about EDN as a biomarker for asthma diagnosis is limited. EDN is easy to sample, can be quantified reliably and stored long‐term^3^ and thus has the methodological qualities to serve as a biomarker. To our knowledge, longitudinal studies of EDN in relation to asthma or wheezing in preschool children are lacking.

In our “Gene Expression in Wheezing and Asthmatic Children*”* longitudinal cohort, we enrolled 156 cases (≥6 months to ≤3 years) at the paediatric emergency ward at Astrid Lindgren's Children's Hospital, Sweden, between 2008 and 2012 when visiting with acute wheeze. The cases came to a first revisit 3 months later, followed prospectively until age 7. In parallel, 102 age‐matched healthy controls were recruited^5^ (Figure 1). Asthma diagnosis at age 7 was based on the global initiative for asthma (GINA) guidelines, as previously described.^6^ Allergen‐specific IgE antibodies (ImmunoCAP™fx5 and ImmunoCAP™Phadiatop) were measured, and allergic sensitization was defined as allergen‐specific IgE ≥ 0.35 kUA/L. EDN levels were measured in serum samples using a microarray‐based semi‐quantitative research assay at Thermo Fisher Scientific, Uppsala, Sweden.

**FIGURE 1:**
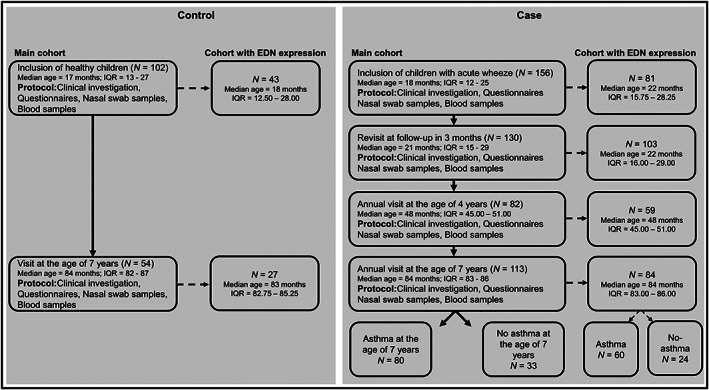
Overview of the study design

The 95% confidence intervals were considered for statistical analysis. Mainly, *t*‐test and *Mann–Whitney U test* were used to measure the central tendency according to their distribution. Analysis of variances was made by ANOVA. All statistical analyses were performed in R (https://www.r‐project.org/).

At the acute visit, no significant differences in EDN levels were observed between preschool wheezers (cases, *n* = 81) and healthy controls (controls, *n* = 43, *p* = .288), or between cases later diagnosed with asthma, cases not diagnosed with asthma, and controls (Figure 2A). At age 7, significantly higher EDN levels were observed in cases than in controls (*p* = .043, Figure 2B). Moreover, at age 7, cases with asthma (*n* = 60) had significantly higher EDN levels than cases without asthma (*p* = .033), and controls (*p* = .017), while no significant difference was observed between nonasthmatic cases and controls (*p* = .461). Increased EDN in cases compared with controls at age 7 was mainly due to the higher levels in children who had developed asthma (Figure 2B). In the longitudinal analysis, cases with asthma at age 7 showed a gradual increase in EDN from the acute visit until age 7 than cases without asthma. From age 4, the difference was significant (age 4: *p* = .021; age 7: *p* = .033, Figure 2C, D).

**FIGURE 2:**
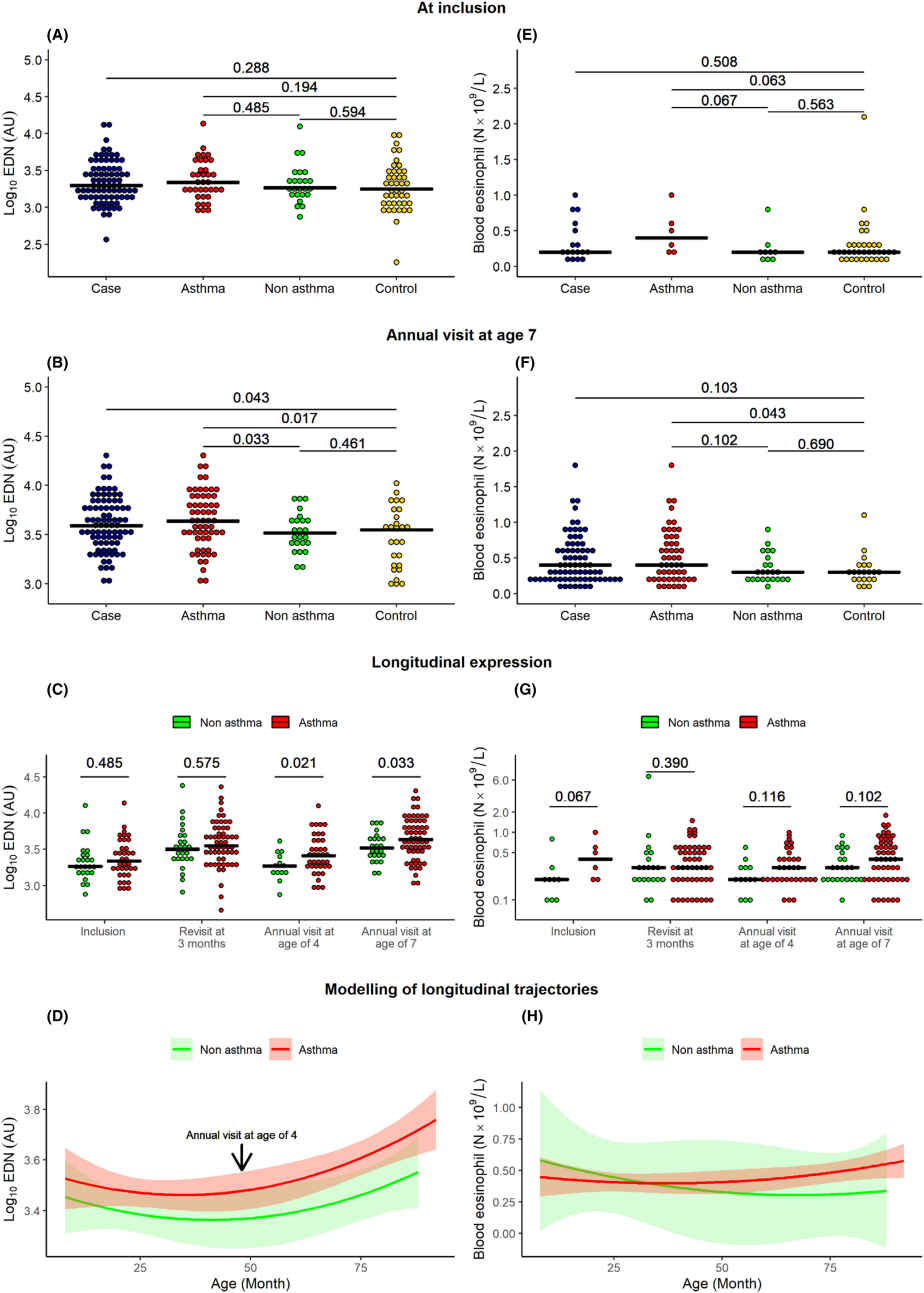
We measured the EDN levels as arbitrary fluorescence intensity units (AU) in children with preschool wheeze (Cases) and healthy children (Controls) (A) at inclusion and (B) at age 7. The cases were also subdivided into asthma (preschool wheezers that had developed asthma at age 7) and nonasthma (preschool wheezers that had not developed asthma at age 7) according to their diagnosis at age 7. (C) Longitudinal EDN levels (AU) in asthma compared with nonasthma at inclusion, at the first revisit after 3 months, and at age 4 and age 7. EDN levels showed a significant (*p* < .05) difference between asthma and nonasthma from the annual visit at age 4. (D) Modelling of longitudinal trajectories of EDN levels. (E–H) Corresponding analysis of blood eosinophil counts (N x 10^9^/L) are shown (E) at inclusion, (F) at age 7 and (G) longitudinally. (H) Modelling of longitudinal trajectories of blood eosinophil counts. Log_10_EDN (AU) follows a significant normal distribution (Shapiro–Wilk normality test, *p* = .169). Thus, we used a t‐test for statistical analysis of EDN. *Mann–Whitney U test was used for the* statistical analysis of blood eosinophil counts.

As eosinophils are the primary producers of EDN, we analysed blood eosinophil (B‐Eos) count at each time‐point and observed a positive correlation between EDN levels and B‐Eos count (Spearman's *ρ* = 0.57, *p* = 1.4 × 10^−26^). However, no significant difference between cases and controls was observed (Figure 2E, F). At inclusion, the B‐Eos count tended to be higher in cases that developed asthma than in those that did not (*p* = .067), but the difference was not longitudinally significant (Figure 2G, H). EDN levels showed slightly higher accuracy than B‐Eos count for predicting asthma status from age 4 (AUC 0.690 vs. 0.647) onwards in the ROC analysis.

For clinical parameters, we compared cases with and without asthma diagnosis at age 7. No significant difference was found in proportion of respiratory syncytial virus (RSV) or rhinovirus (RV) infection at inclusion, or in blood cell counts at any time‐point, nor in longitudinal trajectories. Cases with asthma at age 7 were more often IgE‐sensitized to aeroallergens, both at inclusion (*p* = .033) and at age 7 (*p* = .058). At age 7, cases IgE‐sensitized to aeroallergens showed higher EDN levels (*p* = .001) and B‐Eos count (*p* = .027). In contrast, IgE‐sensitization to food allergens did not show any association. Similarly, no significant association between asthma at age 7 and parental asthma or day care attendance was observed. Comparing EDN levels, IgE‐sensitization to aeroallergens and food allergens, day care attendance and parental asthma, we observed that IgE‐sensitization to aeroallergens at inclusion was significantly associated with asthma diagnosis at age 7 (Ancova‐test, *F*‐value = 4.621, *p* = .038), as was the EDN levels at age 7 (Ancova‐test, *F*‐value = 3.607, *p* = .061). Due to the lack of IgE‐sensitization data at age 4, no regression analysis was performed at this age. At age 7, asthma control test scores showed a highly significant difference between children with and without asthma (*p* = 5.0 × 10^−6^), as expected, but no significant difference (Fisher's Exact Test, *p* > .05) in spirometry (FEV%) or medication with leukotriene receptor antagonist (LTRA) or inhaled corticosteroids (ICS) the 24 h preceding any of the visits was observed. LTRA and ICS medication within the last 24 h did not show any difference in serum EDN levels (*p* > .100) at any time‐point, but LTRA treatment at revisit at 3 months showed low B‐Eos count (*p* = .039) and ICS treatment at the age 4 showed higher B‐Eos count (*p* = .009), which indicates LRTA or ICS medication may influence B‐Eos count, but not EDN levels.

At age 7, more than 70% of the cases in our cohort had developed asthma. Importantly, preschool wheezers that later developed asthma tended to have higher EDN levels than those with acute wheeze that did not develop asthma, and from the age of 4 years, the difference was significant. Previously, in the same cohort, we have shown that preschool wheezers that develop asthma were hospitalized more often and for more extended periods during their first year after inclusion, indicating more severe symptoms than preschool wheezers that do not develop asthma.^6^ This is in line with previous studies that showed higher EDN levels in children with more severe asthma symptoms and where infants hospitalized with recurrent wheezing showed higher EDN levels than infants hospitalized with nonrecurrent wheezing.^7^ In adults, higher EDN levels have been associated with current asthma, poor asthma control and asthma treatment during the last year.^8^ Taken together, EDN emerges as a biomarker for asthma and may even predict asthma development in young children with wheeze.

Eosinophils are the primary producers of EDN,^3^ and both B‐Eos counts and EDN levels have been associated with asthma and suggested as biomarkers for asthma. In addition, eosinophils modulate the function of other leukocytes, and EDN promotes the maturation, migration and activation of dendritic cells (DCs). In contrast, the established marker of eosinophilic activation, eosinophil cationic protein (ECP), appears to activate mast cells, resulting in histamine release.^4^ These may at least partly explain the recent findings in adult asthmatics where ECP and EDN showed association with different asthma characteristics, with EDN being associated explicitly with reported asthma attacks, wheezing and breathlessness and ECP with high neutrophil counts.^8^ If the effect of eosinophil activation and degranulation products is more important than the actual B‐Eos numbers, EDN would serve as a more informative biomarker than the B‐Eos count. In our cohort, the B‐Eos count seemed to be influenced by medication, while EDN levels were not. Our finding that EDN level is a better biomarker for asthma development than B‐Eos count is supported by a recent study in adults.^9^

Allergic sensitization in early life is a risk factor for asthma, also seen in our analyses where allergic sensitization to aeroallergens was associated with later asthma. We found an association between EDN levels and allergic sensitization to aeroallergens at age 7 in the cases. In this cohort, we have previously seen an effect of polysensitization on asthma at age 7.^10^

EDN is, in addition to inflammation, involved in the damage of the lung epithelium, mucus hypersecretion and airway remodelling.^11^ Therefore, the higher EDN levels in children developing asthma may reflect ongoing inflammatory processes, airway remodelling and damaged lung epithelium. This may also explain why no significant difference in EDN levels was observed between toddlers with acute wheeze and the age‐matched healthy controls in our study, as remodelling and epithelium damage may appear later in the disease process.

The longitudinal design with annual revisits, including detailed clinical examinations, questionnaires and biological sampling, is a strength of this study. On the contrary, we have limited statistical power due to the limited sample size. Also, a recall bias may exist as the parents completed the questionnaires at annual revisits. Finally, we cannot exclude a selection bias for attending the seven‐year revisit with a higher interest in the children with symptoms.

In conclusion, increased EDN levels in preschool wheezers may be a biomarker for the ongoing process of asthma development, rather than being a biomarker for acute wheezing, and reflect increased eosinophilic activity. EDN appears to be a better biomarker for predicting the asthma development process in preschool wheezers than B‐Eos count and could be used to support or even enable an earlier clinical asthma diagnosis than what is possible today.

## AUTHOR CONTRIBUTIONS

SC and CS conceptualized this study; KSH, AEF, IH, GH and JRK recruited/maintained the longitudinal cohort; HE, AS and NR analysed EDN levels; SC analysed the data; SC and CS prepared the original draft; AEF, IH, AH, AS, NR, GH and JRK reviewed the manuscript; all authors read and approved the final manuscript.

## FUNDING INFORMATION

This work was financially supported by The Swedish Research Council, the Swedish Heart‐Lung Foundation, Karolinska Institutet, Konsul Th C Bergh Foundation, Freemason Child House Foundation in Stockholm, and The Swedish Asthma and Allergy Association's Research Foundation. Jon R Konradsen was supported by Region Stockholm (clinical research appointment). The funding sources did not influence the study design, the collection, the analysis, the interpretation of the data, the writing of the manuscript or the decision to submit the manuscript for publication.

## CONFLICT OF INTEREST

Sandip Chakraborty, Katarina Stenberg Hammar, Anastasia Filiou, Idun Holmdahl, Angela Hoyer and Gunilla Hedlin have no conflicts of interest to declare. Niclas Rydell, Helena Ekoff and Anders Sjölander are employees of Thermo Fisher Scientific. Dr. Konradsen and Dr. Söderhäll report nonfinancial support from Thermo Fisher Scientific during the study.

## ETHICAL STATEMENT

This study has ethical approval from the Regional Ethics Committee at Karolinska Institutet, Stockholm (Dnr 2008/378–31/4 and Dnr 2014/399–31/3). Written and oral information about the study was provided to the parents, and written consent was obtained from parents and/or legal guardians before the survey.

## Data Availability

The data supporting this study's findings are available from the corresponding author upon reasonable request. Additional supporting information about study methods and findings are available in https://doi.org/10.5281/zenodo.6858442 *but have now also been submitted together with the manuscript for the availability of the reviewers*.
